# Contextual and cultural influences on preschoolers’ aesthetic experience: evidence from a situational teaching intervention in China

**DOI:** 10.3389/fpsyg.2026.1907627

**Published:** 2026-09-04

**Authors:** Fei Fang, Xiangyu Li, Lin Yan, Jian Zhu, Hua Qin, Lijiao Du, Qilong Zhang

**Affiliations:** 1 College of Preschool Education, Capital Normal University, Beijing, China; 2 Xiao Baihe Kindergarten, Beijing, China; 3 College of Fine Arts, Capital Normal University, Beijing, China; 4 College of Fine Arts, Nanjing Normal University, Nanjing, Jiangsu, China; 5 College of Education, United Arab Emirates University, AI-Ain, Abu Dhabi, United Arab Emirates

**Keywords:** aesthetic cognition, aesthetic experience, Chinese preschoolers, contextualized learning, situational teaching

## Abstract

**Introduction:**

Contextual and cultural influences play an important role in shaping young children’s learning, meaning-making, and emotional engagement. In early childhood education, art appreciation provides a valuable context for examining how children perceive, interpret, and emotionally respond to culturally and pedagogically structured learning environments. However, empirical evidence remains limited regarding how culturally grounded contextual teaching approaches influence preschoolers’ aesthetic experience. Drawing on situated learning theory and Li Jilin’s situational teaching framework in China, this study examined the effects of a situational teaching intervention on preschoolers’ aesthetic experience in art appreciation.

**Methods:**

A quasi-experimental pretest–posttest control group design was adopted. Sixty preschool children from two intact classes in a public kindergarten in Beijing, China, participated in the study. One class was assigned to the experimental group, and the other served as the control group. Over an eight-week period, the experimental group participated in situational art appreciation activities organized around life-related scenarios, storytelling, role-play, multisensory exploration, and reflective dialogue, whereas the control group received conventional art appreciation instruction. Children’s aesthetic experience was assessed across three dimensions: aesthetic perception, aesthetic understanding, and aesthetic emotion.

**Results:**

Repeated-measures ANOVAs revealed Group × Time interactions for visual perception and verbal expression, *F*(1, 58) = 8.12, *p* = 0.01, *ηp*^2^ = 0.12; aesthetic understanding, *F*(1, 58) = 9.32, *p* < 0.001, *ηp*^2^ = 0.14; facial expression, *F*(1, 58) = 5.14, *p* = 0.03, *ηp*^2^ = 0.08; and preference explanation, *F*(1, 58) = 4.78, *p* = 0.03, *ηp*^2^ = 0.08. No significant Group × Time interaction was found for viewing duration, *F*(1, 58) = 0.54, *p* = 0.46, *ηp*^2^ = 0.01. After Bonferroni correction, robust intervention effects remained for visual perception and verbal expression and aesthetic understanding, while effects on aesthetic emotion were suggestive and should be interpreted with caution.

**Discussion:**

The results suggest that culturally grounded situational teaching can support preschoolers’ aesthetic-cognitive and socio-emotional engagement by embedding artworks within meaningful, interactive, and emotionally resonant learning contexts. This study provides educational psychological evidence for the role of contextualized teaching in promoting young children’s perception, meaning-making, and emotional expression. It also extends research on contextual and cultural influences by showing how a Chinese situational teaching model may function as a developmentally appropriate contextual learning approach in early childhood art education.

## 1 Introduction

Young children’s learning is not an isolated cognitive process but is shaped by the contextual, social, emotional, and cultural environments in which learning occurs. From an educational psychology perspective, meaningful learning in early childhood depends not only on the presentation of instructional content but also on how children participate in socially mediated activities, connect new information with lived experience, and express cognitive and emotional responses within supportive learning contexts. These contextual and cultural influences are particularly important during the preschool years, when children’s perception, language, emotion, and symbolic understanding develop rapidly.

Art appreciation provides a valuable educational context for examining how young children perceive, interpret, and emotionally respond to structured learning environments. When engaging with artworks, children do not merely identify visual elements; rather, they observe, feel, imagine, interpret, and communicate meaning through verbal, bodily, and emotional responses. In this sense, preschoolers’ aesthetic experience can be understood as a form of aesthetic-cognitive and socio-emotional engagement that involves the integration of perception, meaning-making, and emotion.

Aesthetic experience is therefore not a singular or instantaneous response, but a dynamic psychological process involving the interaction of perceptual, cognitive, and emotional components (Kv and Venukapalli, 2021). Recent advances in neuroaesthetic research have further clarified this multidimensional structure. The “aesthetic triad” model conceptualizes aesthetic experience as an emergent state arising from the coordinated functioning of sensory–motor, emotion–valuation, and meaning–knowledge systems (Chatterjee and Vartanian, 2014). Consistent with this perspective, empirical aesthetics research commonly conceptualizes aesthetic experience as comprising three interrelated dimensions: aesthetic perception, aesthetic understanding, and aesthetic emotion (Leder and Nadal, 2014). This tripartite framework provides a theoretically grounded basis for examining how children engage with artworks in early childhood education.

Despite growing theoretical consensus regarding the multidimensional nature of aesthetic experience, less is known about how culturally grounded contextual teaching approaches shape preschoolers’ aesthetic-cognitive and emotional engagement. In many preschool classrooms, art appreciation activities remain dominated by verbal explanation and image recognition, with limited opportunities for children to explore, interpret, and emotionally engage with artworks through interaction and embodied experience. Such approaches may be insufficient for young children, whose learning is strongly grounded in action, imagination, social interaction, and affective expression. Accordingly, there is a need to examine instructional approaches that can integrate perception, meaning-making, and emotion within developmentally appropriate and culturally meaningful learning contexts.

Recent studies have increasingly examined early childhood visual arts education, children’s aesthetic engagement, and the role of cultural context in art appreciation. For example, classroom-based research in China has shown that comprehensive art education can support young children’s creativity (Huang et al., 2024), while studies in Hong Kong have highlighted continuing challenges in teachers’ visual arts content knowledge and pedagogical implementation (Leung et al., 2025). In empirical aesthetics, cultural match has also been shown to influence art appreciation and viewing behavior among young viewers (Szubielska et al., 2025). However, these studies have not directly examined how a culturally grounded contextual teaching model influences preschoolers’ aesthetic perception, understanding, and emotion. Therefore, further empirical research is needed to clarify how contextual and cultural learning environments shape preschoolers’ aesthetic experience.

Situational teaching offers a useful framework for examining how contextual and cultural influences operate in early childhood learning. Rooted in situated learning theory, situational teaching emphasizes knowledge construction through participation, interaction, and dialogue within meaningful contexts (Lave and Wenger, 1991). This approach also aligns with constructivist and sociocultural theories, which view learning as a socially mediated process shaped by cognitive, emotional, and cultural conditions (Vygotsky, 1978). In art appreciation, situational teaching embeds artworks within life-related scenarios and interactive tasks, thereby transforming children’s engagement from passive observation into contextualized exploration, interpretation, and emotional expression.

In China, Li Jilin’s situational teaching framework provides a culturally grounded model for designing such learning environments. Drawing on the traditional aesthetic idea of the unity of context and emotion, this framework conceptualizes learning as occurring within an “artificially optimized situation” that integrates cognition, emotion, and action (Li, 2018; Wang, 2023). Instruction is organized into four progressive phases: introducing the situation, experiencing the situation, expanding the situation, and recreating the situation. Although situational teaching has been widely discussed as a developmentally appropriate approach in Chinese education, empirical evidence remains limited regarding whether and how this culturally grounded contextual teaching model influences preschoolers’ aesthetic perception, understanding, and emotion in art appreciation.

## 2 Literature review

### 2.1 Aesthetic experience as cognitive–emotional engagement in early childhood

Aesthetic experience is a central concept for understanding how young children engage with artworks. In art appreciation, children are not only exposed to visual forms but also encouraged to observe, feel, interpret, and communicate their responses. Art appreciation has been described as the integration of “understanding and enjoying art” (Seabolt, 2001; Schabmann et al., 2016). Rather than focusing solely on the acquisition of artistic knowledge or technical skills, art appreciation can support children’s broader perceptual, linguistic, emotional, and social development (Bell, 2012). From an educational psychology perspective, preschoolers’ aesthetic experience can therefore be understood as a form of cognitive–emotional engagement in which perception, meaning-making, and affective response are closely intertwined.

Aesthetic perception refers to children’s sensitivity to and engagement with visual and formal features of artworks, such as color, shape, line, space, movement, and composition (Cela-Conde et al., 2004). Research on aesthetic processing suggests that perception is not a passive reception of visual stimuli but an active process involving attention, interpretation, and embodied response (Leder et al., 2004). In preschool children, aesthetic perception is often expressed through looking, pointing, bodily movement, verbal description, and spontaneous affective reactions. These observable responses provide an important foundation for further cognitive elaboration and meaning construction during art appreciation.

Aesthetic understanding involves children’s interpretation of the themes, symbols, narratives, and expressive intentions embedded in artworks (Page, 2022). For young children, such understanding does not usually take the form of abstract formal analysis. Instead, preschoolers often make sense of artworks by connecting visual features with familiar experiences, imagined stories, and emotionally meaningful situations. Empirical studies suggest that children tend to prioritize narrative or thematic content when explaining artworks, reflecting developmentally specific modes of interpretation (Sharples et al., 2003). These findings indicate that aesthetic understanding in early childhood is closely related to children’s symbolic thinking, language development, prior experience, and contextual support.

Aesthetic emotion is another integral dimension of aesthetic experience. It refers to emotional responses that arise during art appreciation and contribute to children’s aesthetic evaluation, preference, and value judgment. Aesthetic emotions do not merely accompany perception and understanding; they also shape how children attend to, interpret, and evaluate artworks (Menninghaus et al., 2019). In preschool contexts, aesthetic emotion is typically expressed through facial expressions, verbal comments, bodily reactions, and explanations of preference. These emotional responses serve as motivational resources that sustain children’s engagement and support deeper interaction with artworks.

Together, aesthetic perception, aesthetic understanding, and aesthetic emotion form an integrated framework for conceptualizing preschoolers’ aesthetic experience. This tripartite structure is consistent with broader empirical aesthetics and neuroaesthetic perspectives, which conceptualize aesthetic experience as involving the coordinated operation of sensory–motor, emotion–valuation, and meaning–knowledge systems (Chatterjee and Vartanian, 2014; Leder and Nadal, 2014). For early childhood research, this framework is particularly useful because it captures the ways in which young children’s aesthetic engagement is simultaneously perceptual, interpretive, and emotional. Recent developmental evidence further suggests that preschoolers’ aesthetic responses are shaped by sensorimotor experience and extend beyond simple liking judgments to include embodied, emotional, and reward-related dimensions (Ardizzi et al., 2023).

### 2.2 Contextual and social influences on preschoolers’ aesthetic meaning-making

Preschoolers’ aesthetic experience does not develop in isolation. Developmental psychology research indicates that young children’s responses to artworks are shaped by developmental stage, prior experience, social interaction, and the learning contexts in which art appreciation occurs. Children’s spontaneous facial, verbal, and bodily responses to artworks reflect early forms of aesthetic judgment, empathy, imagination, and symbolic meaning-making that continue to develop with age and experience (Parsons, 1987). Therefore, the quality of the learning environment plays an important role in shaping how children perceive, interpret, and emotionally engage with artworks.

Contextual support is especially important because preschool children often respond to artworks through concrete experience, embodied action, imagination, and social interaction rather than through abstract verbal reasoning. Learning environments that encourage exploration, interaction, and expression have been shown to enhance children’s participation in art-related activities and support more active forms of aesthetic engagement (Piscitelli and Weier, 2002). In this sense, aesthetic meaning-making is not simply an individual cognitive act but a socially mediated process in which children’s perceptions and emotions are gradually transformed into communicable interpretations.

Teacher–child interaction plays a particularly important role in this process. Through questioning, scaffolding, responsive dialogue, and shared attention, teachers can guide children’s observation, invite emotional expression, and support more elaborated forms of aesthetic understanding (Danko-McGhee, 2006). Compared with one-way explanation, dialogic and interactive approaches are more likely to help children connect immediate perceptual impressions with personal experience, symbolic meaning, and emotional response. Such interaction provides a bridge between children’s spontaneous responses and more reflective forms of aesthetic cognition. Recent museum-based research similarly shows that young children’s aesthetic meaning-making is supported by shared language, sensory materials, embodied responses, and responsive adult–child relationships (Davis and Dunn, 2023).

The assessment of children’s aesthetic experience has also evolved in ways that reflect this contextual and interactive understanding. Early assessment approaches primarily focused on children’s ability to identify and describe formal elements of artworks (Clark et al., 1987). Later approaches incorporated the non-intrusive, unguided Aesthetic Development Interview (ADI) to assess children’s aesthetic growth: researchers transcribe participants’ free responses to artworks, break them into independent thought units, and code these units against the validated 5-stage aesthetic framework (Housen, 2002). More recent studies have emphasized multidimensional assessment tools that can reflect the integrated nature of children’s aesthetic engagement, including perceptual, interpretive, and emotional dimensions (Szubielska et al., 2020).

In the Chinese context, Tu Meiru’s Modern Children’s Art Appreciation Evaluation Scale provides a systematic framework for assessing preschoolers’ aesthetic experience through observable and verbal indicators. The scale operationalizes aesthetic experience across aesthetic perception, aesthetic understanding, and aesthetic emotion, and combines structured observation with interactive assessment procedures (Tu, 2001; Chen, 2023). This approach is suitable for preschool children because it does not rely solely on abstract verbal explanation, but captures children’s aesthetic responses through attention, description, naming, facial expression, preference, and explanation. Accordingly, it provides an appropriate measurement framework for examining how contextualized teaching may influence preschoolers’ aesthetic meaning-making.

### 2.3 Situational teaching as a culturally grounded contextual learning approach

Situational teaching provides a useful framework for examining how contextual and cultural influences operate in preschoolers’ aesthetic learning. Rooted in situated learning theory, it emphasizes knowledge construction through participation, interaction, and dialogue within meaningful contexts (Lave and Wenger, 1991). This view aligns with sociocultural and constructivist perspectives, which regard learning as a socially mediated process shaped by cognitive, emotional, and contextual conditions (Vygotsky, 1978). In art appreciation, situational teaching embeds artworks within life-related scenarios and interactive tasks, transforming children’s engagement from passive observation into contextualized exploration, interpretation, and expression.

Existing art education research has shown that contextual and embodied activities can support children’s aesthetic engagement in multiple ways. Storytelling can help children enter the narrative and emotional world of an artwork, thereby enhancing contextual understanding and imaginative interpretation (Egan, 1989). Role-play allows children to embody characters, actions, and emotional states represented in artworks, supporting both perspective-taking and affective resonance (Szekely, 2015). Multisensory activities can enrich children’s perceptual experience by integrating visual, tactile, auditory, and bodily modalities (Joy and Sherry, 2003). These approaches are particularly appropriate for preschool settings, where learning is predominantly action-based, experience-based, and socially mediated (Vecchi, 2010). Recent research on early childhood visual arts education in Hong Kong has also highlighted continuing challenges in teachers’ content knowledge, pedagogical content knowledge, and classroom implementation, with implications for the wider Asia-Pacific region (Leung et al., 2025).

In China, Li Jilin’s situational teaching framework provides a culturally grounded model for designing contextualized learning environments. Drawing on the traditional aesthetic idea of the unity of context and emotion, this framework conceptualizes learning as occurring within an “artificially optimized situation” designed to integrate cognition, emotion, and action (Li, 2018; Wang, 2023). Instruction is typically organized into four progressive phases: introducing the situation, experiencing the situation, expanding the situation, and recreating the situation. These phases provide a structured pathway through which children can move from initial interest and contextual entry, to embodied experience, extended exploration, and creative or reflective reconstruction.

When applied to preschool art appreciation, situational teaching can be understood not merely as a pedagogical technique but as a culturally grounded contextual learning approach. By connecting artworks with children’s everyday experiences, emotional concerns, bodily actions, and social interactions, it may support the integrated development of aesthetic perception, aesthetic understanding, and aesthetic emotion. However, although situational teaching has been widely discussed in Chinese educational theory and practice, empirical research examining its structured application in preschool art appreciation remains limited, especially studies using experimental designs to test its effects on children’s aesthetic-cognitive and socio-emotional engagement.

The present study addresses this gap by adopting Li Jilin’s four-phase situational teaching framework and applying it to preschool art appreciation activities in a Chinese early childhood education context. By examining whether situational teaching enhances preschoolers’ aesthetic perception, aesthetic understanding, and aesthetic emotion, this study seeks to provide empirical evidence for the role of culturally grounded contextual learning environments in shaping young children’s aesthetic experience. Recent empirical work in China has begun to examine the effects of comprehensive art education on young children’s creativity, suggesting growing interest in classroom-based art interventions in early childhood education (Huang et al., 2024). However, less is known about how culturally grounded contextual teaching shapes preschoolers’ aesthetic perception, understanding, and emotion. Although recent studies have increasingly examined children’s aesthetic engagement, art appreciation, and contextualized learning in early childhood, empirical intervention studies that test culturally grounded contextual teaching models remain limited, particularly in preschool art appreciation contexts.

### 2.4 Current study

Taken together, previous research highlights the multidimensional nature of preschoolers’ aesthetic experience and the importance of contextualized, socially mediated, and emotionally meaningful learning environments. However, limited empirical evidence is available regarding how culturally grounded contextual teaching approaches influence preschoolers’ aesthetic-cognitive and socio-emotional engagement in art appreciation.

**Figure 1:**
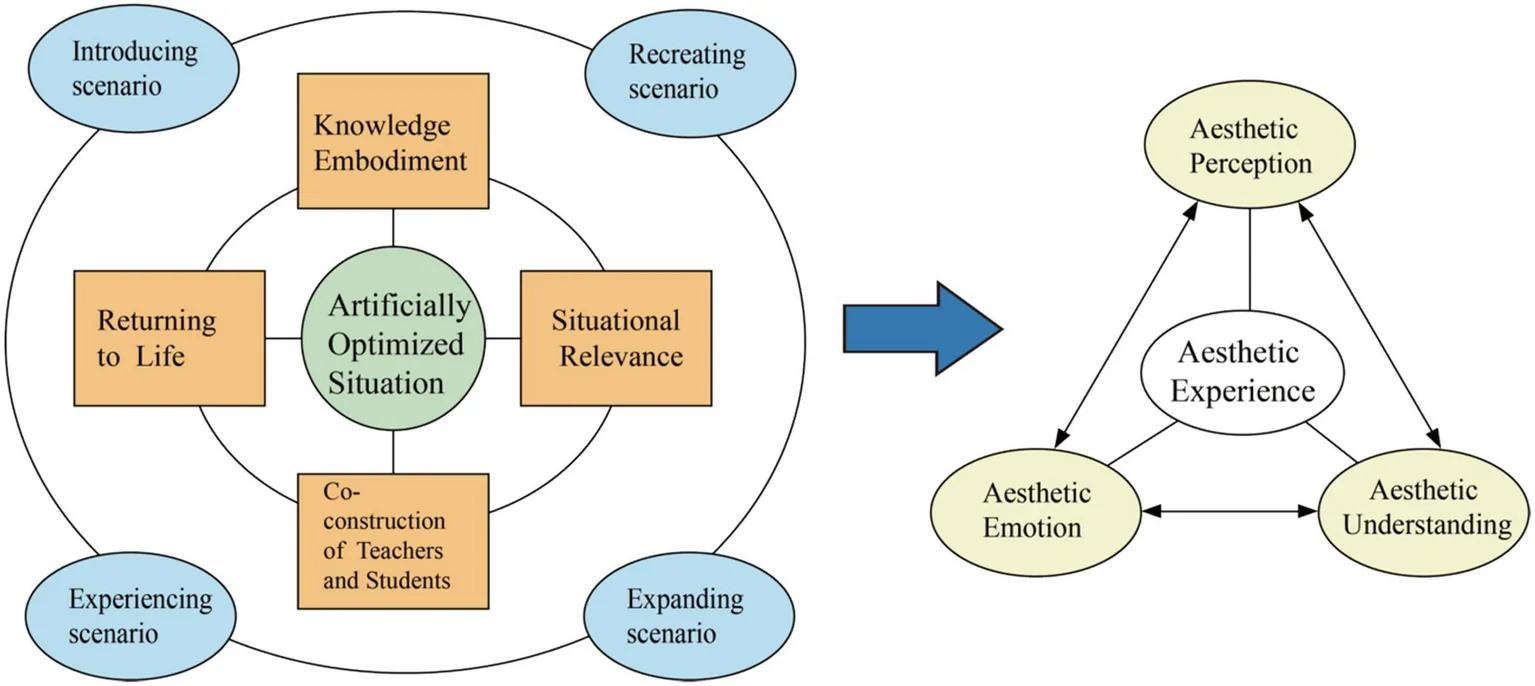
Theoretical framework of the mechanism through which situational teaching influences preschoolers’ aesthetic experience (adapted from Wang, 2023).

To address this gap, the present study adopted Li Jilin’s situational teaching framework and implemented a structured situational teaching intervention in a Chinese preschool context. The intervention embedded art appreciation activities within life-related scenarios and was organized into four progressive phases: introducing, experiencing, expanding, and recreating the situation. Through storytelling, role-play, multisensory exploration, guided observation, and reflective dialogue, the intervention was designed to support the integrated development of aesthetic perception, aesthetic understanding, and aesthetic emotion (see Figure 1).

The theoretical framework guiding this study conceptualizes situational teaching as a culturally grounded contextual learning approach. Through knowledge embodiment, situational relevance, teacher–child co-construction, and connections to everyday life, situational teaching may provide a structured learning context that supports children’s perception, meaning-making, and emotional expression. These three components are conceptualized as interrelated dimensions of preschoolers’ aesthetic experience.

Using a quasi-experimental pretest–posttest control group design, this study examined whether a situational teaching intervention could enhance preschoolers’ aesthetic experience in art appreciation. The following research question guided the study: Can a culturally grounded situational teaching intervention significantly improve preschoolers’ aesthetic perception, aesthetic understanding, and aesthetic emotion in a Chinese early childhood education context? It was hypothesized that preschoolers who participated in contextualized situational teaching would show greater improvements in aesthetic perception, aesthetic understanding, and aesthetic emotion than those receiving conventional instruction.

## 3 Method

### 3.1 Participants

The study was conducted in a public kindergarten located in Haidian District, Beijing. Among the 173 children enrolled at the kindergarten, two intact classes were selected using cluster sampling, yielding a total sample of 60 preschoolers. One class was assigned to the experimental group (*n* = 30, *M_age_* = 66.4 months, *SD* = 2.87; 56.7% girls), and the other class served as the control group (*n* = 30, *M_age_* = 66.1 months, *SD* = 3.04; 60.0% girls). The age range of participants in both groups was 61–72 months. The kindergarten was selected because it was a public preschool with regular art appreciation activities and administrative support for conducting an eight-week classroom-based intervention. Because the intervention needed to be implemented during regular instructional time, two intact classes were selected as clusters. Individual random assignment was not feasible in this naturalistic preschool setting. To reduce baseline bias, the two classes were compared in terms of age, gender distribution, and pretest scores, and no significant differences were found.

An *a priori* power analysis was conducted using G*Power 3.1 for a repeated-measures ANOVA with a within–between interaction. Assuming a medium effect size (*f* = 0.25), an alpha level of 0.05, statistical power of 0.80, two groups, two measurement points, a correlation of 0.50 among repeated measures, and a non-sphericity correction of 1, the minimum required total sample size was estimated to be 34. The final sample of 60 children exceeded this requirement and was therefore considered adequate for detecting medium-sized Group × Time interaction effects in this classroom-based quasi-experimental study.

Independent-samples *t*-tests and chi-square analyses indicated no significant differences between the two groups in age or gender distribution. Teachers in both classes held equivalent academic qualifications in early childhood education and followed comparable daily schedules throughout the study period. In addition, neither the kindergarten nor the participating families had prior experience with specialised art appreciation programmes, ensuring comparable baseline exposure across groups. Descriptive information for the participants is presented in Table 1.

**Table 1:** Participant characteristics by group.

Variable	Experiment group (*n* = 30)	Control group (*n* = 30)
Age (months), M(*SD*)	66.4 (2.87)	66.1 (3.04)
Age range (months)	61~72	61~72
Gender, n (%)		
Boys	13 (43.3%)	12 (40.0%)
Girls	17 (56.7%)	18 (60.0%)

### 3.2 Research design

The study employed a quasi-experimental pretest–posttest control group design. Situational teaching served as the independent variable, while preschoolers’ aesthetic experience in art appreciation was the dependent variable. Aesthetic experience was operationalised across three dimensions: aesthetic perception (AP), aesthetic understanding (AU), and aesthetic emotion (AE). Because random assignment at the individual level was not feasible in the kindergarten setting, two intact classes were assigned to the experimental and control conditions. The experimental group received a structured situational teaching intervention, whereas the control group participated in regular art appreciation activities following the kindergarten’s standard instructional approach.

### 3.3 Instrument

Preschoolers’ aesthetic experience was assessed using the Modern Children’s Art Appreciation Evaluation Scale (MCAAES) developed by Tu (2001). The scale is designed for preschool children and operationalises aesthetic experience across three dimensions: aesthetic perception, aesthetic understanding, and aesthetic emotion. The scale was selected because it operationalizes preschoolers’ aesthetic experience through observable behavioral and verbal indicators, which are appropriate for young children with limited abstract verbal ability. Data were collected through structured observation combined with semi-structured individual interviews conducted during art appreciation activities. The structure and behavioral indicators of the scale are presented in Table 2.

**Table 2:** Structure and indicators of the Modern Children’s Art Appreciation Evaluation Scale (MCAAES).

Dimension	Indicator	Assessment description
Aesthetic perception	Engagement duration and reactions	Duration of focused attention on the artwork and observable behavioral reactions
	Visual perception and verbal expression	Ability to describe visual elements, content, and details of the artwork in response to guided questions
Aesthetic understanding	Naming	Ability to assign a meaningful title to the artwork and justify the choice
Aesthetic emotion	Facial expression	Observable emotional expressions displayed during art appreciation
	Preference and explanation	Stated preference for the artwork and the coherence of the accompanying explanation

All indicators are rated on a 3-point scale (1 = poor, 2 = moderate, 3 = excellent).

The aesthetic perception dimension assesses children’s engagement with artworks, including attention duration, visual perception, and verbal expression. Aesthetic understanding is evaluated through children’s ability to assign an appropriate title to the artwork and explain its meaning. Aesthetic emotion captures children’s emotional responses to artworks, including facial expressions of affect and stated preferences accompanied by explanations.

All items were rated on a 3-point behaviourally anchored scale ranging from 1 (poor), 2 (moderate), to 3 (excellent), with higher scores indicating more advanced aesthetic experience (see Appendix A). The MCAAES has been widely used and validated in Chinese preschool contexts. In the present study, the scale demonstrated good internal consistency (Cronbach’s *α* = 0.81) and high interrater reliability (ICC = 0.92).

#### 3.3.1 Intervention design

Six well-known artworks were selected for the study based on four criteria: visual appeal, relevance to preschool children’s everyday experiences, diversity in artistic styles and historical periods, and inclusion of both representational and abstract works. To avoid learning effects, two artworks—Picasso’s Guernica and Miró’s Harlequin’s Carnival—were used exclusively for the pretest and posttest assessments, while four additional artworks were used during the intervention sessions (see Appendix B).

The intervention was implemented over an eight-week period, with one 45-min session conducted each week. The key difference between the two conditions was the systematic use of contextualized scenarios and interactive tasks in the experimental group. The experimental group received situational art appreciation instruction designed according to Li Jilin’s four-phase situational teaching model: introducing the situation, experiencing the situation, expanding the situation, and recreating the situation. During each session, teachers created life-related scenarios connected to the artworks and guided children through structured activities including storytelling, role-play, guided observation, discussion, and multisensory exploration.

Children participated in both individual and small-group tasks aimed at encouraging active engagement, interpretation, and expressive response to the artworks. Instructional activities were intentionally designed to support the integrated development of aesthetic perception, aesthetic understanding, and aesthetic emotion. In contrast, the control group participated in regular art appreciation activities following the kindergarten’s routine instructional approach, which primarily involved teacher explanation and basic observation without structured situational design.

#### 3.3.2 Procedure

Both the experimental and control groups viewed the same set of artworks during the study period. In the control group, art appreciation sessions were conducted using conventional instructional approaches, primarily involving multimedia presentations of artworks accompanied by brief teacher-led explanations and whole-class discussions. These sessions emphasized visual observation and basic verbal responses without the systematic use of situational tasks.

In contrast, the experimental group participated in situational teaching activities structured according to Li Jilin’s four-phase model, including introducing the situation, experiencing the situation, expanding the situation, and recreating the situation. Instruction incorporated storytelling, role-play, multisensory exploration, and task-based activities designed to promote children’s active engagement with artworks across perceptual, interpretive, and emotional dimensions.

All sessions in both groups were conducted by the class teachers during regular instructional time and followed the same schedule and duration. To support implementation fidelity, the intervention procedures were discussed with the class teachers before the study, and all sessions were video-recorded for later checking. Photographs and field notes were also collected to document children’s observable behaviors, verbal responses, and emotional expressions during art appreciation activities.

### 3.4 Data collection and analysis

Pretest and posttest assessments of preschoolers’ aesthetic experience were administered to both the experimental and control groups using the Modern Children’s Art Appreciation Evaluation Scale (MCAAES). All assessments were conducted individually in a quiet room within the kindergarten and were video recorded to support reliable scoring.

Statistical analyses were performed using SPSS 26.0. Prior to hypothesis testing, independent-samples *t* tests and chi-square analyses were conducted to examine baseline equivalence between the two groups in terms of age, gender distribution, and pretest scores on aesthetic perception, aesthetic understanding, and aesthetic emotion. No significant group differences were identified at baseline.

To examine the effects of situational teaching, repeated-measures analyses of variance (ANOVAs) were conducted separately for each dimension of aesthetic experience, with time (pretest vs. posttest) as the within-subject factor and group (experimental vs. control) as the between-subject factor. Significant Group × Time interaction effects were followed by simple effects analyses to further interpret differences between groups across time points. Effect sizes were reported using partial eta squared (*ηp^2^*). Statistical significance was initially set at *p* < 0.05. Because five related indicators of aesthetic experience were analyzed separately, Bonferroni correction was applied to the five Group × Time interaction tests, yielding a corrected significance threshold of *α* = 0.01. Interpretations of intervention effects were based on this corrected threshold.

### 3.5 Assumption checks

Prior to inferential analyses, the data were screened for missing values, potential outliers, and the assumptions required for repeated-measures ANOVA. Potential outliers were inspected using standardized scores and boxplots. Normality was evaluated through Shapiro–Wilk tests and inspection of skewness and kurtosis values, and homogeneity of variance was assessed using Levene’s tests. No serious violations were identified that would invalidate the use of repeated-measures ANOVA. Because the within-subject factor included only two levels, the assumption of sphericity was not applicable.

## 4 Results

### 4.1 Baseline comparability between the experimental and control classes

**Figure 2:**
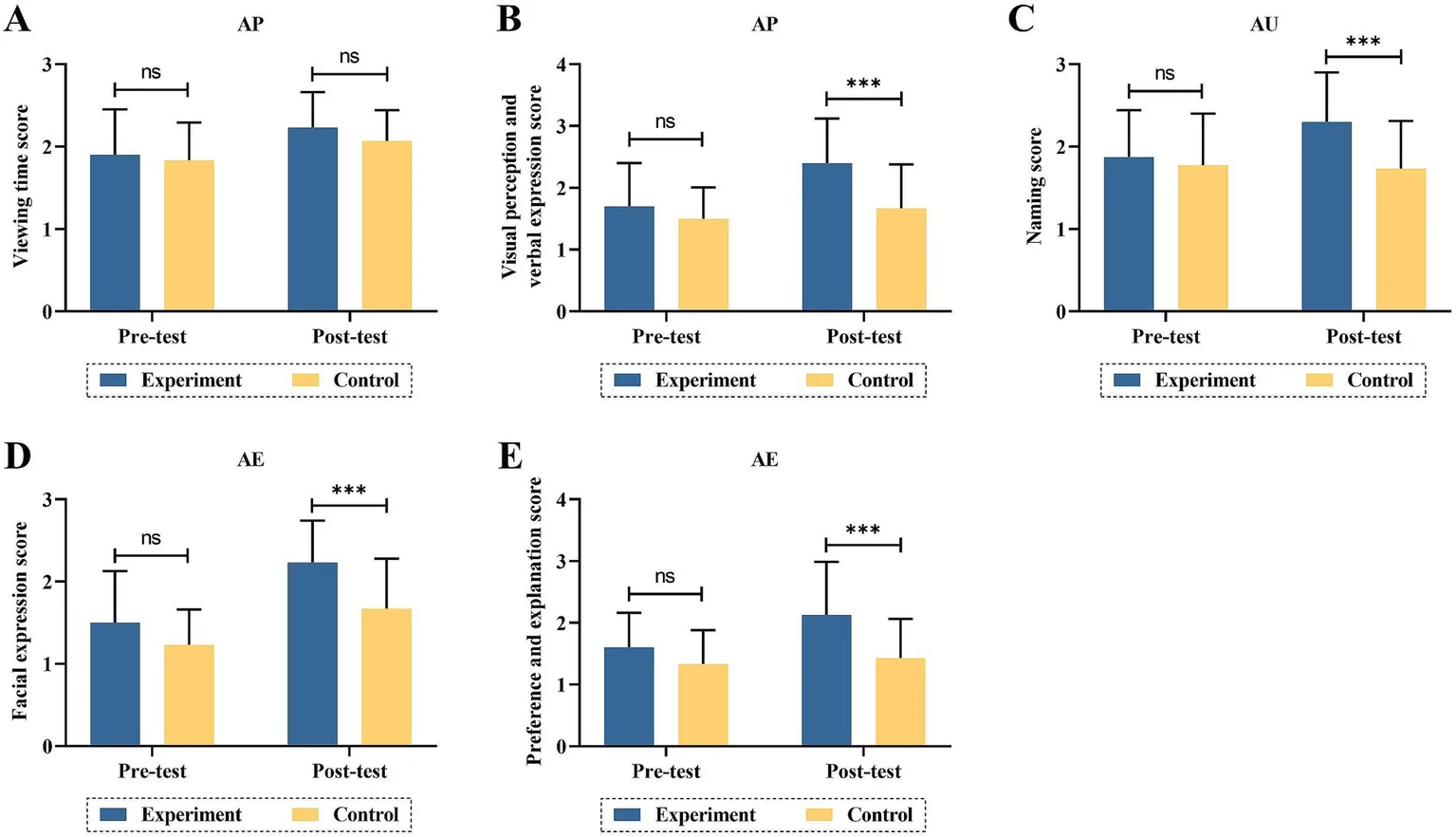
Means and standard deviations of aesthetic experience scores at pretest and posttest for the experimental and control classes. Significance markers indicate uncorrected posttest group comparisons. Interpretations of intervention effects were based on Bonferroni-corrected Group × Time interaction tests.

Independent-samples *t* tests were conducted to assess baseline equivalence between the experimental and control classes. The results indicated no significant pretest differences between the two groups across all dimensions of aesthetic experience, including aesthetic perception, aesthetic understanding, and aesthetic emotion (all *ps* > 0.05). Descriptive statistics for pretest and posttest scores are presented in Figure 2, indicating comparable baseline performance prior to the intervention.

### 4.2 Effects of situational teaching on aesthetic experience

To examine the effects of situational teaching on preschoolers’ aesthetic experience of art appreciation, repeated-measures ANOVAs were conducted for each dimension, with time (pretest vs. posttest) as the within-subject factor and group (experimental vs. control) as the between-subject factor. The results are presented in Table 3.

**Table 3:** Results of repeated-measures ANOVAs for aesthetic experience dimensions.

Dimensions	Item	Data sources	*F*	*p*	*η_p_^2^*
Aesthetic perception	Viewing time	Group	1.47	0.23	0.03
		Time	17.42	<0.001	0.23
		Group × Time	0.54	0.46	0.01
	Visual perception and verbal expression	Group	10.40	<0.001	0.15
		Time	21.45	<0.001	0.27
		Group × Time	8.12	0.01	0.12
Aesthetic understanding	Naming	Group	6.27	0.02	0.10
		Time	6.85	0.01	0.11
		Group × Time	9.32	<0.001	0.14
Aesthetic emotion	Facial expression	Group	11.07	<0.001	0.16
		Time	77.74	<0.001	0.57
		Group × Time	5.14	0.03	0.08
	Preference and explanation	Group	12.09	<0.001	0.17
		Time	10.21	<0.001	0.15
		Group × Time	4.78	0.03	0.08

All F tests were conducted with numerator df = 1 and denominator df = 58. η_p_^2^ represents effect size. Because five Group × Time interaction tests were conducted for related indicators of aesthetic experience, Bonferroni correction was applied to these interaction effects, yielding a corrected significance threshold of α = 0.01. Interpretations of intervention effects were based on this corrected threshold.

For viewing time, the main effect of time was significant, *F*(1, 58) = 17.42, *p* < 0.001, indicating that children in both groups spent more time viewing artworks at posttest than at pretest. However, neither the main effect of group, *F*(1, 58) = 1.47, *p* = 0.23, nor the Group × Time interaction, *F*(1, 58) = 0.54, *p* = 0.46, was significant.

For visual perception and verbal expression, significant main effects of group, *F*(1, 58) = 10.40, *p* < 0.001, and time, *F*(1, 58) = 21.45, *p* < 0.001, were observed. Importantly, the Group × Time interaction was also significant, *F*(1, 58) = 8.12, *p* = 0.01, indicating differential changes over time between the two groups. Simple effects analyses showed no significant difference between the experimental and control classes at pretest, whereas at posttest the experimental class scored significantly higher than the control class.

For naming, the repeated-measures ANOVA revealed significant main effects of group, *F*(1, 58) = 6.27, *p* = 0.02, and time, *F*(1, 58) = 6.85, *p* = 0.01. The Group × Time interaction was also significant, *F*(1, 58) = 9.32, *p* < 0.001. Simple effects analyses indicated that the two classes did not differ significantly at pretest, whereas at posttest the experimental class demonstrated significantly higher naming scores than the control class.

For facial expression, both the main effect of group, *F*(1, 58) = 11.07, *p* < 0.001, and the main effect of time, *F*(1, 58) = 77.74, *p* < 0.001, were significant. The Group × Time interaction was significant at the uncorrected level, *F*(1, 58) = 5.14, *p* = 0.03. However, this interaction did not remain significant after Bonferroni correction.

Similarly, for preference and explanation, significant main effects of group, *F*(1, 58) = 12.09, *p* < 0.001, and time, *F*(1, 58) = 10.21, *p* < 0.001, were observed. The Group × Time interaction was significant at the uncorrected level, *F*(1, 58) = 4.78, *p* = 0.03, but did not survive Bonferroni correction.

To control for the increased risk of Type I error due to multiple comparisons, Bonferroni correction was applied to the five Group × Time interaction tests, resulting in a corrected significance threshold of *α* = 0.01. Under this corrected threshold, the Group × Time interactions for visual perception and verbal expression and naming remained statistically significant, whereas the effects on facial expression and preference explanation did not remain significant after correction.

## 5 Discussion

The present study examined how a culturally grounded situational teaching intervention influenced preschoolers’ aesthetic experience in art appreciation. The findings revealed a differentiated pattern: after Bonferroni correction, robust intervention effects remained for children’s visual perception and verbal expression and aesthetic understanding, whereas the effects on facial expression and preference explanation were significant only at the uncorrected level and should be interpreted cautiously. No significant effect was found for viewing duration. This pattern suggests that the contribution of situational teaching lies not in simply extending children’s observable attention to artworks, but in improving the quality of their aesthetic-cognitive and socio-emotional engagement. From an educational psychology perspective, the findings indicate that contextualized and culturally meaningful learning environments can shape how young children perceive visual information, construct meaning, and express emotional responses.

### 5.1 Contextualized learning and cognitive elaboration in preschoolers’ aesthetic engagement

The first important finding was that children in the experimental group showed greater improvement in visual perception and verbal expression than those in the control group. This suggests that situational teaching supported children not only in noticing visual features of artworks, but also in organizing, elaborating, and communicating their perceptions. In conventional art appreciation activities, children’s responses may remain at the level of object identification or simple repetition of teacher explanations. In contrast, situational teaching embedded artworks within stories, role-play, games, multisensory activities, and life-related tasks, thereby providing concrete contexts through which children could connect visual information with familiar experience and expressive action.

This finding is consistent with situated learning theory, which emphasizes that learning occurs through participation in meaningful social and contextual practices rather than through decontextualized information reception (Lave and Wenger, 1991). It also aligns with sociocultural perspectives that view children’s learning as mediated by interaction, language, and shared activity (Vygotsky, 1978). In the present study, contextualized activities may have helped children transform abstract visual elements such as color, line, shape, rhythm, and composition into meaningful perceptual and verbal responses. Through this process, children were not simply “looking at” artworks, but were actively elaborating visual information within a structured learning context.

The non-significant effect on viewing duration further clarifies this mechanism. Situational teaching did not make children look longer at artworks; rather, it changed how children engaged with what they saw. Preschoolers may be able to extract salient visual information within a relatively short period of time. However, deeper aesthetic engagement requires opportunities to interpret, communicate, and emotionally respond to that information. This result is consistent with previous research suggesting that meaningful art learning depends less on prolonged looking and more on embodied response, dialogic participation, and active meaning-making (Piscitelli and Weier, 2002; Hubard, 2007). Therefore, the present study highlights the importance of participation quality rather than attention duration in preschoolers’ aesthetic experience.

### 5.2 Socially mediated meaning-making in Preschoolers’ aesthetic understanding

The second important finding was that situational teaching significantly improved children’s aesthetic understanding, as reflected in their performance on the naming task. Naming an artwork requires more than recognizing isolated objects or visual elements. It involves organizing perception, identifying relationships among visual cues, connecting them with prior experience, and constructing an interpretable meaning. The higher posttest performance of the experimental group suggests that situational teaching helped children move from surface-level observation toward more integrated and meaningful interpretation.

This improvement may be explained by the socially mediated structure of situational teaching. In the intervention, teacher questioning, guided observation, peer interaction, role-play, and reflective dialogue were not simply classroom activities; they functioned as forms of social mediation. Through these processes, children’s immediate perceptual impressions were gradually transformed into verbalized aesthetic understanding. Teachers scaffolded children’s attention to meaningful visual details, invited them to explain their responses, and encouraged them to connect artworks with personal experiences, emotions, and imagined situations. Such interaction provided a bridge between spontaneous response and communicable interpretation.

This interpretation is consistent with developmental theories of children’s art understanding. Parsons (1987) emphasized that children’s interpretation of artworks develops gradually and is closely related to cognitive development, accumulated experience, and the ability to coordinate perceptual and symbolic meanings. Page (2022) similarly conceptualized aesthetic understanding as a process through which individuals interpret meaning, intention, and expressive content. In early childhood, such understanding is unlikely to emerge through abstract formal analysis alone. Instead, it depends heavily on contextual support, social interaction, and opportunities for expression. The present findings extend this line of research by providing quasi-experimental evidence that a structured contextual teaching intervention can support preschoolers’ early aesthetic meaning-making.

### 5.3 Culturally grounded emotion–cognition integration in situational teaching

Although the intervention showed uncorrected improvements in facial expression and preference explanation, these effects did not remain significant after Bonferroni correction. Therefore, the findings regarding aesthetic emotion should be interpreted cautiously as preliminary evidence that requires replication with larger samples. Children in the experimental group appeared to show richer emotional expressions and provided more elaborated explanations of their preferences after the intervention. This suggests that situational teaching created emotionally meaningful contexts that enabled children to respond to artworks with greater affective involvement and clearer emotional articulation.

This finding is important because aesthetic emotion is not a secondary outcome of art appreciation, but a core component of aesthetic experience. Aesthetic emotions shape attention, preference, evaluation, and meaning-making (Menninghaus et al., 2019). In preschool children, emotional engagement is often expressed through facial expression, bodily response, verbal comment, and preference choice. By connecting artworks with everyday experiences, bodily actions, imagined situations, and interpersonal interaction, situational teaching may have made artworks more emotionally accessible and personally meaningful for young children.

The four-phase structure of Li Jilin’s situational teaching framework may help explain this effect. The phases of introducing, experiencing, expanding, and recreating the situation provide a progressive pathway through which children first enter a meaningful context, then participate in embodied and emotional experience, extend their interpretation, and finally reconstruct or express meaning. This structure reflects the integration of situation, emotion, and action in Chinese situational teaching. Rather than separating cognitive understanding from emotional experience, situational teaching organizes perception, feeling, action, and reflection into a unified learning process.

This mechanism resonates with neuroaesthetic perspectives on aesthetic experience. The aesthetic triad model suggests that aesthetic experience emerges from the interaction of sensory–motor, emotion–valuation, and meaning–knowledge systems (Chatterjee and Vartanian, 2014). Research on embodied and affective responses to art also highlights the role of motion, emotion, and empathy in aesthetic experience (Freedberg and Gallese, 2007). In the present study, children were encouraged to perceive artworks, enter imagined situations, enact roles, discuss feelings, and reconstruct meanings. This integrated process may have supported the coordination of perceptual, interpretive, and emotional components of aesthetic experience. Thus, the effect of situational teaching lies not in increasing attention time, but in promoting emotion–cognition integration within a developmentally appropriate learning context.

### 5.4 Contextual and cultural contributions to Educational Psychology

The findings of this study also have broader implications for educational psychology research on contextual and cultural influences. Situational teaching, particularly in Li Jilin’s framework, is not merely a set of instructional techniques. It can be understood as a culturally grounded contextual learning model that integrates cognition, emotion, action, and lived experience. By designing “artificially optimized situations,” teachers create learning environments in which children can participate, interact, imagine, and construct meaning. This model provides a culturally embedded example of how contextualized learning can be organized in early childhood education.

The present study contributes to research on contextual influences by showing that children’s aesthetic experience is shaped by the structure and quality of the learning environment. When artworks were presented through teacher explanation and basic observation, children’s responses remained comparatively limited. When artworks were embedded in meaningful situations involving storytelling, role-play, multisensory exploration, and reflective dialogue, children demonstrated greater gains in visual-verbal expression, meaning construction, and emotional response. These results suggest that contextualized teaching can support children’s cognitive elaboration and socio-emotional engagement by linking visual stimuli with personal experience, social interaction, and culturally meaningful forms of expression.

The study also contributes to research on cultural influences by demonstrating the potential of a Chinese situational teaching tradition as an educational psychological model. Rather than treating culture as a background variable, this study shows how a culturally grounded instructional framework can structure children’s learning processes. The unity of context and emotion in Chinese situational teaching provides a meaningful theoretical basis for designing learning environments that support preschoolers’ perception, understanding, and emotion. In this sense, the findings extend educational psychology research by illustrating how culturally embedded pedagogical traditions may contribute to broader theories of contextualized learning and child development.

### 5.5 Summary and implications

In summary, the present study provides empirical evidence that culturally grounded situational teaching can support aspects of preschoolers’ aesthetic experience in art appreciation. Its robust effects were reflected not in longer viewing duration, but in improvements in visual-verbal expression and meaning construction, while changes in emotional expression and preference explanation were suggestive and should be interpreted with caution. This differentiated pattern suggests that contextualized teaching may be especially effective in improving the quality and depth of young children’s aesthetic-cognitive and socio-emotional engagement.

Theoretically, this study contributes to educational psychology in three ways. First, it shows that contextualized learning can support cognitive elaboration by helping preschoolers transform visual information into verbal expression and meaningful interpretation. Second, it highlights the role of socially mediated meaning-making, showing how teacher questioning, peer interaction, guided observation, and reflective dialogue can help children convert immediate perceptual and emotional responses into communicable aesthetic understanding. Third, it demonstrates that culturally grounded situational teaching can support emotion–cognition integration by organizing children’s perception, feeling, action, and reflection within structured learning situations.

Practically, the findings suggest that early childhood art appreciation should move beyond teacher explanation and image recognition toward more interactive, contextualized, and emotionally meaningful forms of learning. Teachers can support preschoolers’ aesthetic experience by designing life-related scenarios, using storytelling and role-play, incorporating multisensory exploration, and creating opportunities for reflective dialogue. Such practices may help children connect artworks with their own experiences, express visual and emotional responses, and construct more meaningful interpretations.

The study also has implications for understanding contextual and cultural influences in early childhood education. By drawing on Li Jilin’s situational teaching framework, this study illustrates how a Chinese pedagogical tradition can function as a culturally embedded contextual learning model. The findings suggest that culturally meaningful instructional frameworks may contribute not only to local educational practice, but also to broader educational psychology theories concerning how learning environments shape children’s cognition, emotion, and meaning-making.

Several limitations should be acknowledged. First, although the sample size exceeded the minimum requirement indicated by the *a priori* power analysis, the participants were recruited from two intact classes in one public kindergarten in Beijing. Therefore, the findings should be interpreted as preliminary evidence from a localized classroom-based intervention and should not be generalized to the broader preschool population without caution. Future studies should include larger samples from multiple kindergartens, regions, and sociocultural contexts. Second, the intervention lasted 8 weeks, and the study did not examine long-term retention effects. Future research could extend the intervention duration and include follow-up assessments to determine whether improvements in aesthetic experience are sustained over time. Third, although the present study used structured observation and individual interviews, future research may incorporate additional process-level measures, such as eye-tracking, behavioral coding, or age-appropriate physiological indicators, to further examine the psychological mechanisms through which contextualized teaching influences children’s aesthetic engagement.

Overall, this study suggests that situational teaching can function as a developmentally appropriate and culturally meaningful contextual learning approach in early childhood education. By integrating perception, meaning-making, and emotion within structured social and cultural contexts, situational teaching offers a promising pathway for enriching preschoolers’ aesthetic experience and for advancing research on contextual and cultural influences in educational psychology.

## 6 Conclusion

This study examined whether a culturally grounded situational teaching intervention could improve preschoolers’ aesthetic perception, aesthetic understanding, and aesthetic emotion in a Chinese early childhood education context. The findings generally supported the research hypothesis. Compared with conventional instruction, situational teaching showed robust effects on children’s visual perception and verbal expression and meaning construction. Effects on emotional expression and preference explanation were observed at the uncorrected level but did not remain significant after Bonferroni correction and should therefore be interpreted cautiously. In addition, the intervention did not significantly increase viewing duration, suggesting that the intervention primarily improved the quality and depth of children’s aesthetic engagement rather than the amount of observable attention.

These results suggest that situational teaching may function as a culturally meaningful contextual learning approach that supports young children’s aesthetic-cognitive and socio-emotional development. By integrating contextualized cognition, socially mediated meaning-making, and emotion–cognition integration, the study contributes to educational psychology research on how contextual and cultural learning environments shape preschoolers’ engagement with artworks. Given the localized sample and quasi-experimental design, the findings should be interpreted as preliminary evidence and require replication in larger and more diverse preschool contexts.

## Data Availability

The original contributions presented in the study are included in the article/supplementary material, further inquiries can be directed to the corresponding authors.
