# Supplementary material for: Contextual and cultural influences on preschoolers’ aesthetic experience: evidence from a situational teaching intervention in China
Source: Front Psychol. 2026 Sep 4;17:1907627. doi: 10.3389/fpsyg.2026.1907627 (PMC13585757; doi:10.3389/fpsyg.2026.1907627)
Supplement: Supplementary file 1 [file Supplementary_file_1.docx]

APPENDIX A Modern Children’s Art Appreciation Evaluation Scale (MCAAES).

| Dimension | Item | Poor (1 point) | Medium (2 points) | Excellent (3 points) |
| --- | --- | --- | --- | --- |
| Aesthetic perception | Viewing time | Briefly glances at the artwork and quickly shifts attention away. | Maintains visual attention on the artwork for less than one minute. | Sustains visual attention on the artwork for more than one minute. |
|  | Visual perception and verbal expression | Identifies the main object or general content of the artwork with minimal description. | Describes the main content and simple relationships between visual elements. | Provides detailed descriptions of the main content, specific visual details and the overall composition of the artwork. |
| Aesthetic understanding | Naming | Assigns a title that refers to a single, isolated element in the artwork. | Assigns a title that corresponds to the content described by the child. | Assigns a title that reflects the content and conveys meaning, creativity, or humorous interpretation. |
| Aesthetic emotion | Facial expression | Displays a neutral expression similar to that shown during routine reading activities. | Displays observable interest (e.g.  smiling, focused attention). | Displays strong emotional engagement (e.g., excitement, surprise, expressive facial reactions). |
|  | Preference and explanation | Expresses a general preference with little reference to feelings or reasons. | Identifies key elements (e.g., objects or themes) and provides basic reasons, with limited emotional expression. | Expresses clear emotional responses and provides detailed explanations using age-appropriate art-related language (e.g., color, shape, composition, or theme). |

APPENDIX B The Intervention Procedure.

| Artwork | Activity title | Teaching procedure |
| --- | --- | --- |
| The Sleeping Gypsy | The Gypsy Girl’s Wonderful Journey | **P1:** The teacher establishes a narrative scenario in which children visit "Grandpa Rousseau's" home and discover a mysterious painting, arousing curiosity. Children are invited to prepare a short drama entitled *Visiting Grandpa Rousseau's Home*.  **P2:** Through a guided storytelling activity, children closely observe the artwork and express their perceptions, imagination, emotions, and understanding by creating stories based on the painting.  **P3:** Children engage in role-play, embodying the characters and animals in the painting to explore emotional states and deepen their understanding of the artwork's content and form.  **P4:** Following the drama performances, the teacher and children jointly reflect on their experiences of task completion and aesthetic appreciation. |
| Broadway Boogie Woogie | Adventure in the Kingdom of Grids | **P1:** The teacher initiates an outdoor hopscotch game to introduce the concept of grids and stimulate interest.  **P2:** Indoors, a play-based adventure and treasure-hunt scenario is created. Children search for "treasures" while attending to the artwork's visual structure and composition.  **P3:** Music is introduced to support multisensory exploration, helping children perceive rhythmic similarities between the artwork and musical patterns.  **P4:** Children and the teacher share reflections on the treasure-hunt experience, guided by both the artwork's rhythm and the accompanying music. |
| Eight Horses | Galloping Horses | **P1:** The teacher introduces the song *Horse Racing*, allowing children to perceive imagery and emotional qualities conveyed through music.  **P2:** Children role-play as horses, exploring the artwork's formal elements and emotional expressions through movement.  **P3:** Using pony stickers and music videos, children imagine themselves as galloping horses, physically expressing the dynamic movement and energy depicted in the artwork.  **P4:** Through embodied movement and discussion, children consolidate their understanding of the artwork's dynamic form and emotional intensity. |
| Winding the Skein | A Gift for Mom | **P1:** The teacher introduces colorful yarns to create interest. Children work in pairs to role-play a mother-daughter thread-winding scenario and are given the task of creating a drawing as a gift for their mother.  **P2:** Children engage in tactile exploration by winding yarn into balls, experiencing texture and movement.  **P3:** Building on this tactile experience. the teacher guides children to observe the artwork's composition, colors, lines. And emotional expressions conveyed through facial features.  **P4:** Children connect the artwork to their own life experiences and emotions toward their mothers and create a personal painting as an emotional response. |

Note. (P1 = Introducing the Situation, P2 = Experiencing the Situation, P3 = Expanding the Situation, P4 = Recreating the Situation)
